# Applications of functionally-adapted hydrogels in tendon repair

**DOI:** 10.3389/fbioe.2023.1135090

**Published:** 2023-02-02

**Authors:** Jiacheng Hu, Shen Liu, Cunyi Fan

**Affiliations:** ^1^ Department of Orthopedics, Shanghai Sixth People's Hospital Affiliated to Shanghai Jiao Tong University School of Medicine, Shanghai, China; ^2^ Shanghai Engineering Research Center for Orthopaedic Material Innovation and Tissue Regeneration, Shanghai, China

**Keywords:** hydrogel, tendon repair, functionally-adapted, stem cell, macrophage

## Abstract

Despite all the efforts made in tissue engineering for tendon repair, the management of tendon injuries still poses a challenge, as current treatments are unable to restore the function of tendons following injuries. Hydrogels, due to their exceptional biocompatibility and plasticity, have been extensively applied and regarded as promising candidate biomaterials in tissue regeneration. Varieties of approaches have designed functionally-adapted hydrogels and combined hydrogels with other factors (e.g., bioactive molecules or drugs) or materials for the enhancement of tendon repair. This review first summarized the current state of knowledge on the mechanisms underlying the process of tendon healing. Afterward, we discussed novel strategies in fabricating hydrogels to overcome the issues frequently encountered during the applications in tendon repair, including poor mechanical properties and undesirable degradation. In addition, we comprehensively summarized the rational design of hydrogels for promoting stem-cell-based tendon tissue engineering *via* altering biophysical and biochemical factors. Finally, the role of macrophages in tendon repair and how they respond to immunomodulatory hydrogels were highlighted.

## 1 Introduction

**FIGURE 1:**
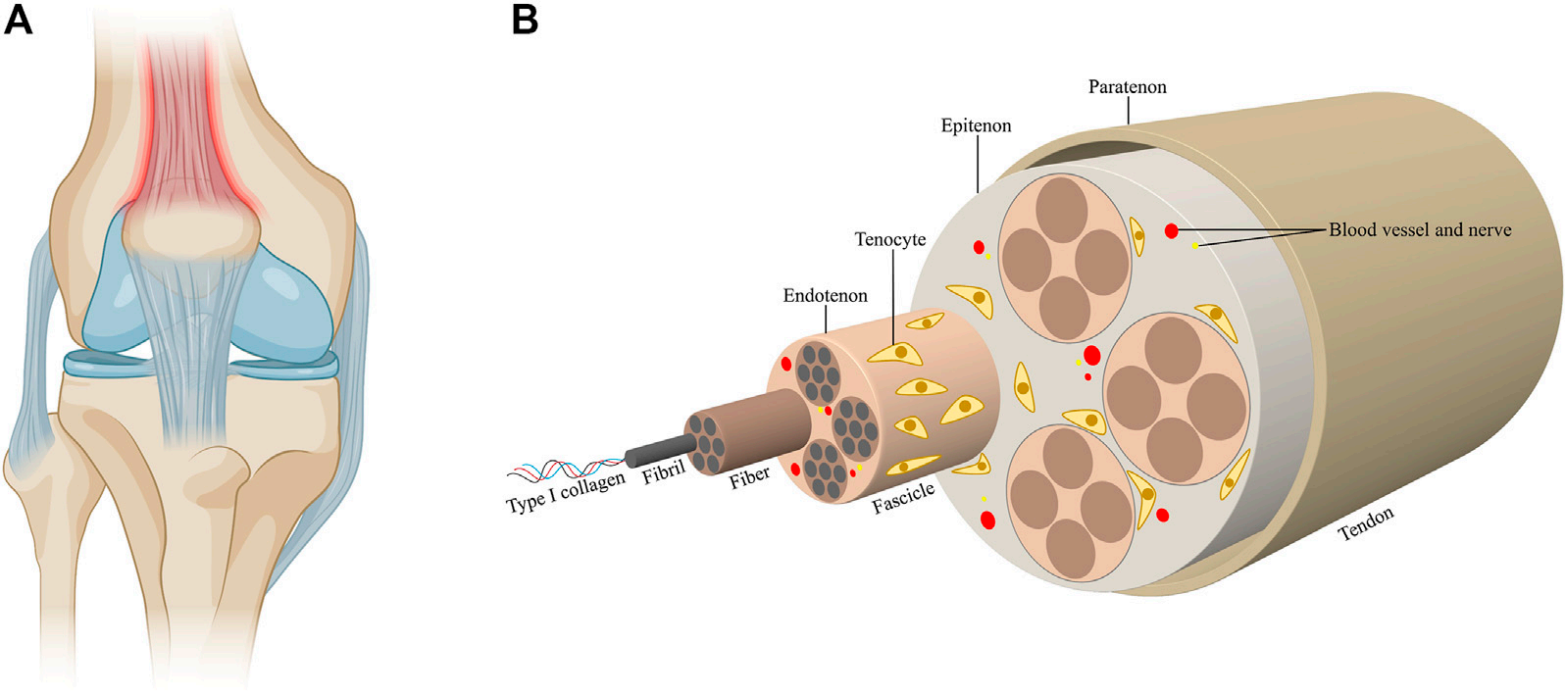
Tendon structure and composition. **(A)** Tendons are connective tissues that connect muscle to bone. **(B)** Triple-helical type I collagen molecules aggregate to form collagen fibrils, which further assemble into fascicles. The fascicles combine to form tendon tissues. Fibers are ensheathed by the endontenons, while the tendons are surrounded by the epitenon, which is further surrounded by the paratenon. Tenocytes sparsely align between the collagen fibers. The blood vessels and nerves accompany the collagen fibers in the tendons.

Tendons are dense fibrous connective tissues that connect muscle to bone (Figure 1A). Tendons are predominantly composed of highly aligned collagen type I fibrils with tenocytes sparsely aligned between the collagen fibers, while other components of tendons include elastin, glycoproteins, proteoglycans and other collagen types (Thorpe and Screen, 2016; Voleti et al., 2012; Millar et al., 2021). Tendon tissues have a hierarchical arrangement of collagen molecules. Triple-helical type I collagen molecules aggregate to form bundles of twisted collagen fibrils, which further assemble into fascicles, the tendon units, to form a structure with high tensile strength, thus endowing tendons with the function of transmitting the forces of muscular contractions to bones (Zhang et al., 2005; Nourissat et al., 2015a; Citeroni et al., 2020a) (Figure 1B). Tendon injuries could result in the disruption of tissue integrity and impairment of load-bearing capacity. The spectrum of tendon injuries includes acute tendon rupture and chronic tendinopathy (Nourissat et al., 2015a). While tendon injuries are usually associated with excessive mechanical overuse, environmental factors and certain genetic factors also contribute to the development of tendon injuries. Tendon injury has gradually become prevalent among people of all ages, thereby causing detrimental effects on their daily life (Millar et al., 2021). However, current treatments available for tendon injuries still face severe challenges. The efficacy of conservative therapies including anti-inflammatory drugs, exercise-based therapy and injection of autologous growth factor remain controversial (Khan and Scott, 2009; Zhou and Wang, 2016; Irby et al., 2020; Millar et al., 2021). Simultaneously, invasive strategies which are accompanied by multiple postoperative complications increase the occurrence of tendon re-rupture (Voleti et al., 2012; Zhang et al., 2021a). With the advances in material science, biomaterials have been broadly employed in the repair of tendon tissue, representing promising strategies to ameliorate clinical outcomes (No et al., 2020a). The failure of conventional strategies in treating tendon injuries could mainly be attributed to the hypocelluarity and hypovascularity properties of tendon tissues. Biomaterials, either applied alone or in combination with growth factors, stem cells or gene regulators, could mimic native tendon architectures and induce appropriate biological response. The applications of biomaterials also offer the researchers an approach to manipulate the cellular and molecular events during tendon repair by altering the designing of the biomaterials (Freedman et al., 2022a; Tang et al., 2022). Current therapeutic platforms for tendon repair mainly include fibrous membranes, hydrogels and decellularized materials (No et al., 2020a; Zhang et al., 2021a). Fibrous membranes have been the most extensively studied and applied biomaterials for tendon repair, as they mimic the natural structures of tendon extracellular matrix (ECM) environment (Freedman and Mooney, 2019a). However, it has been suggested that a single approach would not be sufficient to meet the various phases of tendon repair. Therefore, combining different strategies to fabricate multifunctional biomaterials would be pivotal in achieving complete functional recovery of tendon injury.

Hydrogels are emerging materials with three-dimensional network structures of hydrated polymeric chains (Drury and Mooney, 2003; Vermonden et al., 2012). With high water content of over 90% of weight, hydrogels possess properties and structures analogous to ECM, providing a good platform for cellular growth and tissue regeneration (Yue et al., 2015; Naahidi et al., 2017). In addition, the porous structure of hydrogel allows it to carry drugs, growth factors and cells, therefore making it a promising platform for the delivery of drugs or biomolecules. By altering network structures and bindings between drugs and polymer chains, the delivery of drugs by hydrogels could be manipulated at a desired rate (Hoare and Kohane, 2008; Li and Mooney, 2016a). Given their excellent biological properties, adjustability of their physiochemical characteristics and similarity to native ECM, hydrogels hold great promise in the applications of tendon repair and regeneration (No et al., 2020a; Yao et al., 2022). However, hydrogels still have multiple drawbacks that limit their practical applications. Firstly, hydrogels exhibit poor mechanical properties due to their high water content. As tendons are load-bearing tissues with high strength and toughness, conventional hydrogels are still not strong enough to mimic or replace human native tendons (Hua et al., 2021; Freedman et al., 2022b). Secondly, the high load and deformation in practical use would result in the loss of structural integrity in hydrogels, further resulting in undesirable degradation and unstable release of drugs or other therapeutic agents (Cai et al., 2022). To this regard, the ideal functionally-adapted hydrogels designed for tendon repair are suggested to meet the following criteria: high biocompatibility without triggering detrimental immune responses, sufficient mechanical properties to withstand the high load, controllable degradation behaviors, desirable structures for cell proliferation and tissue regeneration and on-demand delivery of loaded biologics or cells.

In this review, we discuss the latest advances in functionally-adapted hydrogels in tendon repair. We will first describe the complicated process of tendon repair and healing. Then we will discuss recent studies on tuning mechanical properties and degradation properties of hydrogels for tendon repair. Finally, we will focus on how the behavior of stem cell and macrophage polarization could be modulated by various material properties of hydrogels.

## 2 Tendon repair and healing

**FIGURE 2:**
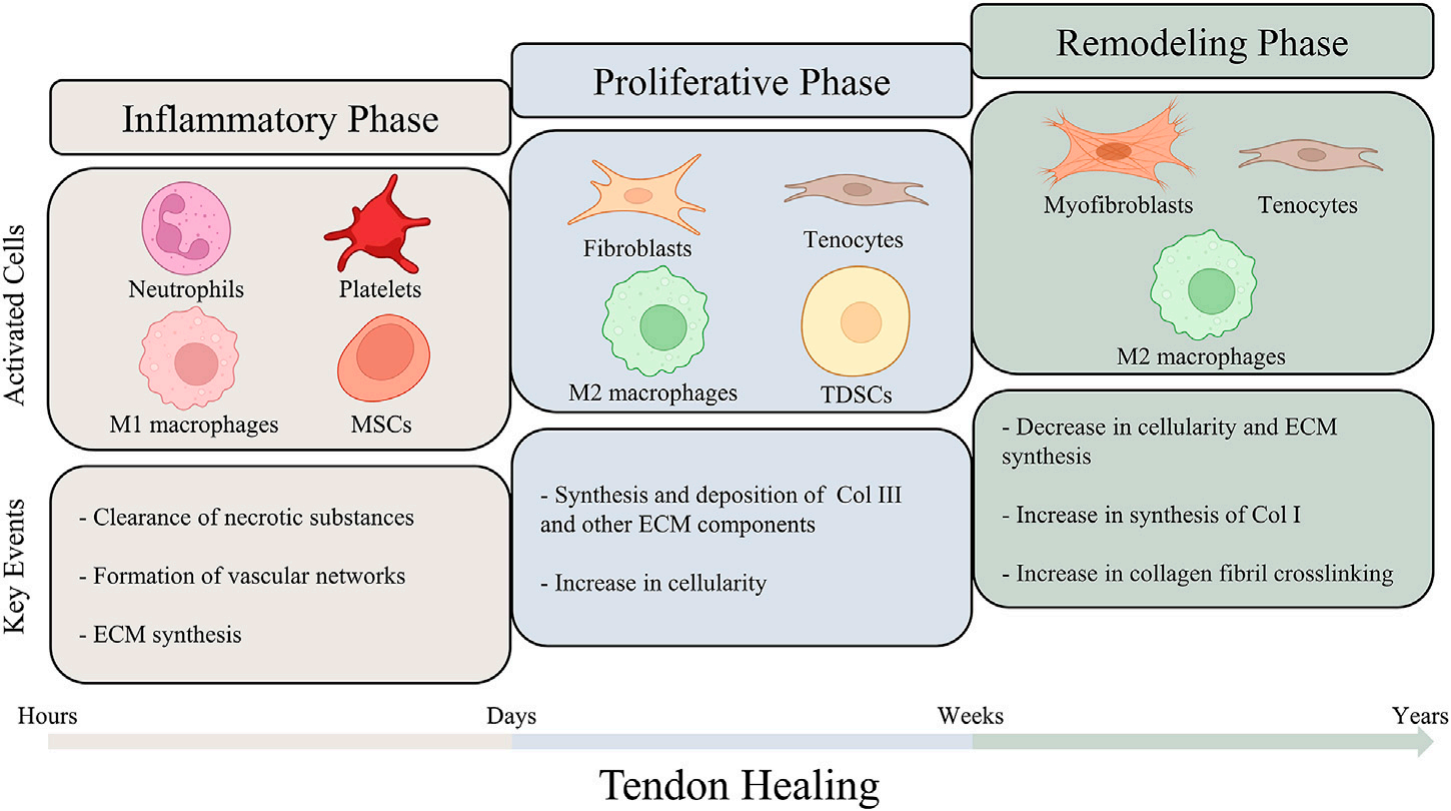
A schematic illustration of the different phases of tendon healing. The process of tendon healing could be divided into three overlapping phases: inflammatory phase, proliferative phase and remodeling phase. As tendon healing progresses, different types of cells are activated and lead to sequence of key events. The figure also summarizes the estimated durations of the three phases.

Due to the lack of biomechanical, histopathological and molecular researches on human tendons, the mechanisms under tendon healing still remain unclear. It is generally considered that the process of tendon healing goes through three overlapping phases, including the inflammatory phase, the proliferative phase and the remodeling phase (Hope and Saxby, 2007; Voleti et al., 2012; Wu et al., 2017a). These three phases are regulated by different molecules and cells compartments, and the duration of each phase depends on the site and severity of injury (Docheva et al., 2015; Millar et al., 2021; Russo et al., 2022a) (Figure 2).

The inflammatory phase represents the initiation of the tendon healing, in which circulating inflammatory cells, such as neutrophils and monocytes/macrophages, are attracted by the platelet-derived cytokines and infiltrate into the injury site (Leong et al., 2020; Hou et al., 2021). Among all the inflammatory cells involved in the inflammatory phase, macrophages have been considered to play an essential role in modulating the healing process. Macrophages could be principally segmented into two functional sub-phenotypes, including pro-inflammatory M1 phenotypes and anti-inflammatory M2 phenotypes (Murray Peter et al., 2014; Sunwoo et al., 2020). Recent studies have been focusing on the regulation of macrophage polarization by biomaterials to enhance tendon healing (Ye et al., 2021). M1 macrophages play a dominant role in the inflammatory phase. The inflammatory cells clear up the cellular debris and secrete angiogenic factors to promote blood supply. Furthermore, cytokines are secreted to recruit resident cells adjacent to the injury site, which start to synthesize ECM components and initiate tissue repair. (Docheva et al., 2015; Arvind and Huang, 2021; Hou et al., 2021). While persistent inflammatory damage could result in poor healing and chronic tendinopathy, a proper early-stage inflammatory response is essential for triggering tendon repair (Thomopoulos et al., 2015; Arvind and Huang, 2021).

Following the inflammatory phase, the proliferative phase is characterized by synthesis of ECM components and lasts for several weeks. Fibroblasts derived from sheath and synovium, and tenocytes derived from epitenon and endotenon are recruited to the injury site and begin to proliferate (Hope and Saxby, 2007; Leong et al., 2020). Type III collagen and other ECM components are excessively synthesized and deposit randomly on the injury site, therefore contributing to early matrix production (Zhang et al., 2021a). Tendon-derived stem cells (TDSCs) are also activated in this phase of healing. A recent study employing single-cell transcriptomics and lineage tracing identified *Tppp3* ^*+*^ *Pdgfra* ^*+*^ cells as potential tendon stem cells, which were demonstrated to be capable of generating tenocytes and self-renewing after tendon injury (Harvey et al., 2019). A diversity of cytokines, including basic fibroblast growth factor (bFGF), transforming growth factor beta (TGF-β) and insulin-like growth factor-1 (IGF-1), are continuously released by cells and tissues at the injury site (Nourissat et al., 2015b). These cytokines functionally augment cellular proliferation and angiogenesis, thereby giving support to sufficient ECM production (Docheva et al., 2015; Titan et al., 2019; Leong et al., 2020). At this stage of tendon healing, the previous M1 macrophages are increasingly polarized towards the M2 phenotype, playing a role in ameliorating inflammation and decreasing scar formation (Sugg et al., 2014; Sunwoo et al., 2020; Hou et al., 2021).

In the final phase of tendon healing, the amount and metabolism of tenocytes and fibroblasts gradually reduce, leading to a decrease in the synthesis of ECM components and cellularity. In contrast to the diminished ECM synthesis, the synthesis of type I collagen increases and type I collagen subsequently replaces type III collagen. The tenocytes and the collagen fibers are previously randomly aligned in the injury site. In the remodeling phase, they begin to reorient in accordance with the direction of tension forces. Furthermore, more crosslinks are formed within the collagen fibers, endowing the regeneration tissue with tensile strength (Hope and Saxby, 2007; Nourissat et al., 2015b; Lomas et al., 2015; Leong et al., 2020; Hou et al., 2021). However, the repaired tendon could hardly achieve the mechanical capacity of normal tendon tissues, as certain scar tissue would remain permanently (Walden et al., 2017; Titan et al., 2019).

In the process of tendon healing, two different ways of cellular healing have been purposed: intrinsic healing and extrinsic healing (Voleti et al., 2012; Zhang et al., 2021a). Intrinsic healing is dominated by cells from tendon and epitenon, which is associated with scarless healing and better recovery from the injury. Extrinsic healing is controlled by fibroblasts and inflammatory cells migrated from the peripheral sites, which are related to the formation of scar tissues and adhesions (Sharma and Maffulli, 2006; Docheva et al., 2015; Zhao et al., 2015; Wu et al., 2017b; Legrand et al., 2017; Liu et al., 2021). Optimal tendon repair would be achieved by the coordination between both mechanisms throughout the three stages of tendon healing. The first two stages are characterized by extrinsic healing to form fibrovascular scar, while the intrinsic healing gradually dominates the healing process, which replaces the scar tissue and further contributes to better biomechanical characteristics and fewer complications for the regenerated tendons. However, in tendon injury, the lack of cellularity and vascularity of tendons would lead to relatively low capacity for intrinsic healing (Yin et al., 2010; Abbah et al., 2014). Therefore, the excessive activation of extrinsic healing would result in multiple complications following tendon repair, such as tendon adhesions, tendon re-rupture and failed repairs (Dy et al., 2012; Voleti et al., 2012).

In summary, the complicated process of tendon repair and healing is modulated by numerous cellular, molecular and environmental components. While great advances have been made in the understanding of tendon healing, how to target the key factors in the healing process and achieve functional tendon repair still remains a severe challenge. Hydrogels, with their similarity to native ECM, could provide favorable substrates for tendon healing and precise targeted delivery of biologics into the repair site, therefore exhibiting promising potential in the therapeutics of tendon injury.

## 3 Influence of hydrogels properties on tendon repair

### 3.1 Tuning mechanical properties of hydrogels for tendon repair

**FIGURE 3:**
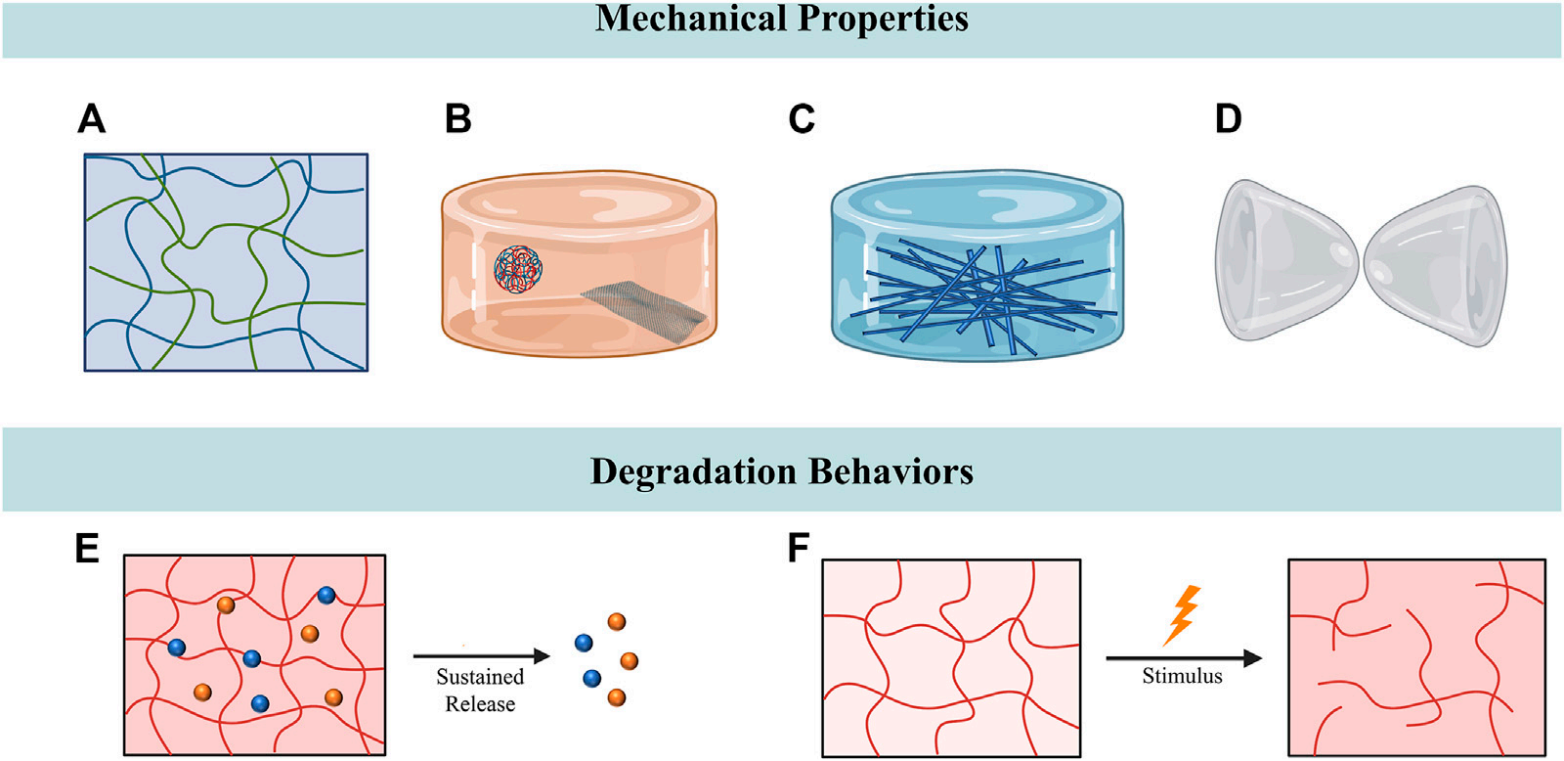
Tuning mechanical properties and degradation behaviors for tendon repair. **(A)** Interpenetrating hydrogels composed of two polymer networks with enhanced mechanical stability. **(B)** Nanocomposite hydrogels comprised hydrogel and nanometre-sized fillers. **(C)** Impregnating the fibrous constructs with hydrogels to fabricate fiber-reinforced hydrogels. **(D)** Hydrogels with self-healing properties could maintain intact structures after being ruptured. **(E)** Hydrogel-based sustained release system. **(F)** The degradation of responsive hydrogels could be manipulated by stimulus.

Tendons are continuously subjected to mechanical loadings transmitting from bones to muscles in daily life. Under the circumstance of tendon injury, the mechanical stimulation generated by mobilization has been suggested to enhance tendon repair by inducing the tenogenic differentiation of stem cells and regulating the cell behaviors of tendons (Subramony et al., 2013; Morais et al., 2015; Sheng et al., 2020). Therefore, mobilization therapy has been considered to bring benefits to patients with tendon injuries by improving tendon healing, increasing tensile strength and reducing tendon adhesions (Rrecaj et al., 2014). Although the hydrogel systems have been widely used for tendon repair, the lack of sufficient mechanical toughness and strength has restricted their applications. Conventional hydrogels are not capable of providing sufficient mechanical support for tissue regeneration and are prone to breaking up due to the friction between tendon and sheath (Allur Subramanian et al., 2022; Cai et al., 2022). Multiple strategies have been utilized in hydrogel fabrication to mimic the mechanical properties of native tendon tissues, including interpenetrating hydrogels, hybridization with nanomaterials and self-healing hydrogels (Vedadghavami et al., 2017). Among all the approaches employed to tune the mechanical properties of hydrogels, interpenetrating polymer networks have been the most commonly studied one (Figure 3A). For instance, alginate ionically crosslinked with calcium and covalently crosslinked polyacrylamide were combined to synthesize a tough and stretchable double interpenetrating hydrogel network. This highly stretchable and tough hydrogel was reported to have fracture energies of about 9,000 Jm^-2^ and could be stretched over 20 times its initial length. Due to its exceptional mechanical properties and high crosslinking degree, this tough hydrogel could avoid the uncontrollable drug release and also had a high drug-loading capacity, thus making it a promising choice for drug delivery. The tendon repair effect of the hydrogel was evaluated in the rat model of Achilles tendon rupture and it was observed to hasten tendon healing and reduce scar formation (Sun et al., 2012; Freedman et al., 2022b). To mimic the exceptional mechanical properties of tendon tissues, Park et al. transformed the alginate/polyacrylamide double network hydrogel into a strong and tough anisotropic hydrogel through stretching and subsequent solvent exchange and crosslinking. With the various degree of stretching, the anisotropic hydrogel exhibited elastic modulus ranging from 1.8 MPa to 6.5 MPa. Furthermore, the researchers employed laying/braiding to fabricate hierarchically braided anisotropic hydrogel cables with significantly improved fracture strength (4.7 MPa) compared to the unbraided ones (Park and Kim, 2022). Similarly, Choi et al. reported an anisotropic hydrogel with mechanical properties reminiscent of tendons. A triple network hydrogel was primarily manufactured, which was comprised of ionically cross-linked alginate, covalently cross-linked polyacrylamide, and poly (2-hydroxyethyl aspartamide) modified with aminopropyl imidazole (PHEA-API). The triple network hydrogel further underwent stretching and secondary cross-linking to form a anisotropic structure with a high modulus (3.0 MPa) and strength (0.8 MPa) (Choi et al., 2022). Another approach fabricated a scaffold by integration of two-dimensional nanomaterials to enhance the mechanical properties of gelatin hydrogel (Figure 3B). The gelatin hydrogel was reinforced by the hybridization of polyglycerol-functionalized reduced graphene oxide (PG) and polyglycerol-functionalized molybdenum disulfide (PMoS_2_), which was observed to possess Young’s modulus of 0.5 MPa. In addition to improving the mechanical properties, the incorporation of PG accelerated tendon regeneration, while PMoS_2_ suppressed inflammation during tendon repair. *In vivo* studies also testified the tendon regenerative capability of the scaffold, with improved Achilles functional index, Adhesion Grading System, biomechanical properties of the healed tendons (Barzegar et al., 2022). Parmaksiz et al. designed a nanocomposite hydrogel scaffold originated from decellularized tendons. Hydroxyapatite (HAp) was incorporated into the decellularized bovine tendon hydrogel to enhance the mechanical properties of scaffold. Mechanical tests demonstrated that, after the incorporation of HAp, the scaffolds with 10% HAp exhibited the highest mechanical strength (Parmaksiz, 2022).

Recent studies have focused on the combination of conventional fiber and hydrogel to develop functionally-adapted fiber–hydrogel composites (Figure 3C). Yang et al. reported a multi-layered fiber-hydrogel composite fabricated by co-electrospinning and ultraviolet crosslinking of poly-ε-caprolactone (PCL) and methacrylated gelatin (mGLT). This hybridized scaffold combined the advantages of both PCL and mGLT, as PCL fibers provided the mechanical strength of the tendon and mGLT fibers mimicked the native tendon microenvironment. Mechanical tests revealed that crosslinked multilayer composite scaffolds exhibited significantly higher tensile strength (∼1.55 MPa) than the non-crosslinked ones. The incorporation of human ADSCs into the scaffold demonstrated its capacity in guiding cellular functionality (Yang et al., 2016). Similarly, another study also invented a synthetic fiber-reinforced hydrogel scaffold with stress-strain property and ability to withhold water. The core of this hydrogel scaffold was composed of ultrahigh molecular weight polyethylene (UHMWPE) fibers, which resembled the collagen fibers in tendons. The injectable form of polyvinyl alcohol/gelatin hydrogel was then injected into the UHMWPE fibers and eventually surrounded the fiber bundles to mimic the ECM components surrounding the collagen fibers. This hydrogel scaffold showed tensile yield strength (77.0 ± 5.0 MPa) and yield strain (9.9% ± 1.3%) similar to human Achilles tendon, and higher tensile modulus (1,245 ± 130 MPa) at 30 MPa (No et al., 2020b).

When the hydrogels are injected or implanted into the injury sites, the hydrogels are prone to be displaced or fragmented due to the physical distortion caused by the movement of tendon sliding. Most hydrogels have difficulty in maintaining at the implantation site due to lack of adhesion. The fragmented hydrogels would further lead to detrimental inflammatory response which hampers tendon repair (Freedman and Mooney, 2019b; Cai et al., 2022). In this regard, constant efforts have been dedicated to designing hydrogels with self-healing properties or adhesive strength in recent years (Bertsch et al., 2022) (Figure 3D). Dang et al. reported a multifunctional hydrogel derived from the skin secretion of *Andrias davidianus* (SSAD) with self-healing property, high adhesive strength and adjustable porous structures. SSAD-derived hydrogels could reconnect the ruptured tendons without the fixation of suturing and self-heal after fragmentation in the model of Achilles tendon transection. As hydrogels composed of natural materials, SSAD-derived hydrogels contained extensive amino acids and cytokines that promote tendon healing, and even show exceptional antioxidant and antibacterial abilities. These hydrogels were therefore expected to serve as a scaffold which could both promote tendon healing and prevent peritendinous adhesion (Dang et al., 2022). In another study, Cai et al. developed a self-healing hyaluronic acid (HA) hydrogel which encircled the repaired tendons to prevent the formation of peritendinous adhesions. The HA hydrogel was fabricated by the mixture of aqueous solutions of oxidized HA-containing aldehyde groups and adipic acid dihydrazide-modified HA. When the implanted HA hydrogel ruptured during the tendon sliding, it could maintain the intact structure through the reconstruction of the internal dynamic covalent linking of hydrazine bonds. Experiments demonstrated that, compared to the traditional hydrogel, the application of self-healing hydrogels reduced the infiltration of macrophages, therefore preventing inflammation-induced adhesion. In addition, the self-healing hydrogel was combined with the PCL electrospun nanofibers to form a composite anti-adhesion barrier with satisfying mechanical strength (Cai et al., 2022).

### 3.2 Tuning degradation of hydrogels in tendon repair

All hydrogels would experience *in vivo* degradation after being implanted. Hydrogel degradation tends to have a direct impact on the controlled release of drugs and the eventual outcome of tissue repair. The ideal degradation is required to be synchronized with the rate of cell proliferation and blood vessel infiltration to guarantee tissue regeneration (Mantha et al., 2019; Cao et al., 2021; Xu et al., 2022a). On the other hand, benefiting from its structural porosity, plasticity and biocompatibility, the hydrogel system offers an excellent choice for the targeted delivery of biologics for tissue engineering. The degradation rate of hydrogel would significantly influence the release of biologics. However, under the complicated environment of tendon healing, the precise regulation of hydrogel degradation is difficult to achieve, thus leading to failure of regeneration or undesirable release of biologics. Simultaneously, the appropriate degradation rate of hydrogels still remains elusive (Ashley et al., 2013; Li and Mooney, 2016b; Narayanaswamy and Torchilin, 2019). Therefore, the controllable degradation of hydrogels has been a keen scope of interest in hydrogel design and its applications in tendon repair.

Current researches mainly focus on designing hydrogel as the sustained-released carrier in order to overcome the burst release of biologics or cells at the early stage of implantation (Zhang et al., 2020; Zou et al., 2020) (Figure 3E). Kim et al. demonstrated that sustained release of drugs could be achieved by encapsulating the anti-inflammatory drug celecoxib into the injectable poly (organophosphazene) (PPZ) nanoparticle hydrogel system. *In vivo* experiments showed that the hydrogel system could remain in the injection site for a month, while the drug encapsulated was gradually released consistent with the degradation of hydrogel. The hydrogel system was proven to be effective in alleviating excessive inflammation in chronic Achilles tendinitis and triggering the regeneration of the damaged tendons. *In vivo* tendon regeneration studies revealed enhanced stiffness and tensile strength of the regenerated tendon tissues (Kim et al., 2022). In the terms of controllable degradation of hydrogels, modifying the composition and structure of hydrogel has been the most commonly used strategy (Li et al., 2011). For instance, Qiu et al. reported a polyethylene glycol (PEG)-based hydrogel system with controllable degradation rates, which is accomplished by varying the composition ratio between oligo (poly (ethylene glycol) fumarate) (OPF) and acrylated poly (ethylene glycol)–dithiothreitol (DTT). Higher amounts of OPF or concentrations of DTT would lead to higher degradation rate. This degradable hydrogel system was utilized to deliver mesenchymal stem cells (MSCs) towards the injury site of tendon. The controllable degradation of hydrogel, ranging over nearly 1 month, offered an approach to control the delivery of MSCs to the injured tissue (Qiu et al., 2011).

In recent years, considerable interest has been attracted to hydrogels which are able to respond to external changes, representing a novel approach to regulate the degradation of hydrogels (Koetting et al., 2015; Mantha et al., 2019) (Figure 3F). For instance, Cai et al. developed a metalloproteinase-2 (MMP-2) degradable hydrogel by crosslinking allyl glycidyl ether modified carboxymethyl chitosan with MMP-2 substrate peptide, which was loaded with TGF-β1 siRNA polyplexes. After tendon injury, the hydrogel could respond to the enhanced expression level of MMP-2 in the peritendinous area, thereby providing a method for the on-demand delivery of TGF-β1 siRNA polyplexes to inhibit fibroblasts proliferation and suppress the degree of peritendinous adhesion *in vivo* (Cai et al., 2021). Li. et al. designed a composite hydrogel which was comprised of clay nanoparticles and a biodegradable alginate hydrogel network. The clay nanoparticles served as drug carriers and were embedded in the degradable alginate hydrogel network. The hydrogel network could gradually degrade and respond to the low pH environment around the injury sites, while the clay nanoparticles were expected to be released correspondingly. The composite hydrogel exhibited promising extended releasing properties, with an estimated half-life of over 10 days. The researchers subsequently implanted the composite hydrogel into the Achilles tendon injury model to evaluate its biodegradation and biocompatibility. The composite hydrogel was estimated to be fully degraded within 5 weeks and did not lead to detrimental immune response at the injury site (Li et al., 2018a). Simultaneously, hydrogels with a relatively slow degradation rate could serve as barriers for preventing tendon adhesions as tendon itself heals slowly when compared with other tissues (Kuo et al., 2014). In another study, Martin et al. designed a reactive oxygen species (ROS)-degradable hydrogel by cross-linking the PEG-based hydrogel with ROS-degradable poly (thioketal) (PTK) polymers. This ROS-degradable hydrogel could scavenge exogenous ROS and endogenous ROS of cells, therefore protecting the encapsulated cells from the damage of cytotoxic ROS and promoting the survival of cells. Meanwhile, the gradual degradation of hydrogels guaranteed proper *in vivo* delivery of cells (Martin et al., 2020).

The narrow ranges of mechanical properties and degradation behaviors from current hydrogels appear to be two major problems which have limited their applications to a large extent. Extensive studies have been dedicated to manipulating the mechanical properties and degradation behaviors of hydrogels (Table 1). However, designing hydrogel systems with controlled architecture and multi-functions to meet different application requirements, including on-demand delivery or providing biomimetic microenvironment, in tendon repair is still challenging.

**TABLE 1:** Mechanical properties and degradation behaviors of hydrogels and their performance in tendon repair.

Category		Approach	Outcome
Mechanical Properties	Interpenetrating hydrogels	Hydrogel composed of alginate ionically crosslinked with calcium and covalently crosslinked polyacrylamide	The hydrogel had fracture energies of about 9,000 Jm-2 and could be stretched over 20 times its initial length, which provided mechanical tissue integrity
			Freedman et al. (2022b)
		Alginate/polyacrylamide double network hydrogel	The double network hydrogel were transformed into hierarchically anisotropic hydrogel cables with mechanical properties similar to tendons
			Park and Kim (2022)
		Alginate/polyacrylamide/PHEA-API triple network hydrogel	Transformation into anisotropic tough hydrogels, with a high modulus (3.0 MPa) and strength (0.8 MPa), *via* stretching and crosslinking
			Choi et al. (2022)
	Nanocomposite hydrogels	Gelatin hydrogel reinforced by PG and PMoS_2_	Young’s modulus of the gelatin-based hydrogel increased to 0.5 MPa after addition of PG and PMoS_2_. The regenerated tendons exhibited enhanced mechanical properties and less adhesions
			Barzegar et al. (2022)
		Decellularized bovine tendon hydrogel incorporated with HAp	Enhanced compressive strength after incorporation of HAp
			Parmaksiz (2022)
	Fiber-reinforced hydrogels	Multilayered composite scaffold composed of PCL fibers and mGLT hydrogel network	Crosslinked multilayer composite scaffolds exhibited exceptional tensile strength (∼1.55 MPa)
			Yang et al. (2016)
		UHMWPE fibers impregnated with polyvinyl alcohol/gelatin hydrogel	Tensile yield strength (77.0 ± 5.0 MPa), yield strain (9.9% ± 1.3%) and water content (∼70%) similar to human Achilles tendon. The hydrogel scaffold facilitated the ingrowth of organized collagenous tissue
			No et al. (2020b)
	Self-healing hydrogels	Hydrogel derived from the skin secretion of SSAD	Enhanced biomechanical properties of the regenerated tendons and reduction in peritendinous adhesions
			Dang et al. (2022)
		HA hydrogel with internal dynamic covalent linking of hydrazine bonds	Intact structure of the hydrogel barrier and reduction in peritendinous adhesions
			Cai et al. (2022)
Degradation Behaviors	Sustained-released carrier	Injectable PPZ nanoparticle hydrogel system loaded with celecoxib	Long term anti-inflammatory effects *via* sustained release of celecoxib. Enhanced stiffness and tensile strength of the regenerated tendon tissues
			Kim et al. (2022)
		PEG-based hydrogel composed of varying amounts of OPF and DTT	Delivery of MSCs into the tendon tissue could be manipulated by the amount of hydrogel compositions
			Qiu et al. (2011)
	Responsive hydrogels	MMP-2 responsive hydrogel loaded with TGF-β1 siRNA polyplexes	TGF-β1 siRNA polyplexes were released in MMP-2 overexpression microenvironment. Inhibition of fibroblast proliferation and peritendinous adhesion
			Cai et al. (2021)
		Alginate hydrogel network loaded with clay nanoparticles	Slow and extended degradation triggered by pH
			Li et al. (2018a)
		PEG-based hydrogel crosslinked with ROS-degradable PTK polymers	The degradation was mediated by ROS. The hydrogel scavenged ROS and promoted the survival of encapsulated MSCs
			Martin et al. (2020)

## 4 Applications of hydrogel/stem cell therapy in tendon repair

Due to the hypocellularity of tendon tissues, the natural process of tendon healing undergoes a long period of time and results in the formation of undesired scar tissue, which impedes the functional tendon repair (Sharma and Maffulli, 2005; Yang et al., 2013). In order to improve the quality of tendon healing and ameliorate the complications after tendon repair, biomaterials combined with stem cells are continuously developed to achieve optimal outcomes (Docheva et al., 2015). With capacity to self-renew and differentiate into various cell types, the stem cells are expected to survive and differentiate into tendon cells, eventually regenerating fully functional tendon tissues similar to pre-injury level. Moreover, stem cells are able to promote the proliferation and migration of circumjacent cells through paracrine signaling (Ahmad et al., 2012; Costa-Almeida et al., 2019). Multiple types of stem cell sources have been widely investigated in the differentiation toward tenogenic lineage and regeneration of tendon tissues, including TDSCs, MSCs and amniotic epithelial cells (AECs). MSCs, such as bone marrow mesenchymal stem cells (BMSCs) and ADSCs, are regarded as the most studied stem cell type for tissue engineering, while TDSCs and AECs have also attracted considerable interests in tendon repair (Gulotta et al., 2012; Lin et al., 2018; Costa-Almeida et al., 2019; Citeroni et al., 2020b; Li et al., 2021a; Russo et al., 2022b). Despite the advances in stem-cell-based tendon tissue engineering, the delivery and activation of the transplanted stem cells in the tendon injury site remains unresolved, which would significantly impede the success of transplantation (Lui, 2015). Due to their excellent biocompatibility with stem cells and surrounding tissues, the hydrogels have been extensively employed as carriers for stem cells in tendon repair. Previous efforts have designed hydrogels with controlled degradation properties for cell encapsulation and delivery into the injured tendon tissues (Qiu et al., 2011). Recent studies have also reported that functionally-adapted hydrogels with tunable structures and properties provide favorable substrates for the proliferation and tenogenic differentiation of seeded stem cells (Zhang et al., 2021a; Nakhlband et al., 2021; Xu et al., 2022a). Therefore, in addition to serving as delivery vehicles, the role of hydrogels in regulating stem cell fate has raised the enthusiasm of researchers to design novel hydrogels. Utilizing novel technologies to fabricate hydrogels with desirable biophysical and biochemical factors would benefit the applications of stem cell-based therapies in tendon repair (Table 2).

**TABLE 2:** Tuning biophysical and biochemical factors of hydrogels for stem cells.

Category		Approach	Outcome
Biophysical	Stiffness	Collagen gels ranging from 20–80 kPa	The expression of tendon-related genes of BMSCs peaked at 40 kPa
			Sharma and Snedeker (2010)
		PEG-based hydrogels ranging from 10–90 kPa by altering monomer concentrations	Higher stiffness PEG-based hydrogels promoted the scleraxis expression of human MSCs
			Rehmann et al. (2016)
		Tuning crosslink density in gelatin hydrogels	Gelatin hydrogels with high stiffness promoted the proliferation of TDSCs and formation of F-actin stress fibers. Hydrogels with low stiffness promoted the tenogenic differentiation of TDSCs
			Liu et al. (2018)
		Tuning polymer concentration and crosslink density in alginate hydrogels	TPCs exhibited higher expressions of *Scleraxis* and *Col XII* but lower expressions of *Col I* in hydrogels of higher stiffness. More cell spreading was observed in hydrogels of low stiffness
			Marturano et al. (2016)
	Topography	Anisotropic magnetic collagen hydrogel achieved by exposure of iron oxide nanoparticles to external magnetic field	The anisotropic hydrogel directed aligned cellular orientation and enhanced tenogenic differentiation
			Xu et al. (2021)
		Anisotropic gelatin hydrogels fabricated by incorporation of cellulose nanocrystals and exposure to magnetic field	Human ADSCs encapsulated in the anisotropic hydrogel exhibited a spindle-shape morphology and higher expression level of TNC
			Echave et al. (2019)
		Multilayered composite scaffold composed of PCL fibers and mGLT hydrogel network	ADSCs impregnated into the multilayer constructs with increasing metabolic activity and were able to receive exogenous biochemical factors
			Yang et al. (2016)
		Chitosan-based asymmetric hydrogel scaffold	ADSCs impregnated into the multilayer constructs with increasing metabolic activity and were able to receive exogenous biochemical factors
			Yang et al. (2016)
		CS nanowires/alginate composite hydrogels fabricated by 3D printing and mechanical stretching	Composite hydrogels with multiscale structures induced order alignment and differentiation of BMSCs and TDSCs. Promotion of collagen fiber alignment and tendon repair regeneration from bone to tendon
			Ma et al. (2022)
		ADSCs-loaded collagen-fibrin hydrogels incorporated with PLGA scaffolds	The parallel frame structure of the multilayered scaffold was able to mimic the tendon alignment, while the hydrogel promoted the proliferation and tenogenic differentiation of human ADSCs
			Jiang et al. (2020)
		Multilayered scaffolds loaded with pre-differentiated ADSCs generated by 3D bioprinting and melt electrospinning techniques	The 3D-bioprinted multilayered scaffold loaded with pre-differentiated ADSCs mimicked the structure and cell distribution of tendon-to-bone interface. Promotion of tendon-to-bone interface regeneration with better histological score and collagen organization, and similar T2 value to normal enthesis tissue
			Jiang et al. (2022)
	Mechanical Stress	MSCs-loaded aligned dense collagen hydrogels were stimulated under static strain or cyclic rest for 48 h	Elevated expressions of tenogenic marker, *Scx*, and lower expressions of osteogenic and chondrogenic markers, *RUNX2* and *aggrecan*
			Park et al. (2022a)
		GelMA-alginate hydrogels loaded with human BMSCs were stimulated by static mechanical stretching	Mechanical stretching induced cell spreading and elongation, and higher expression levels of collagen I and III
			Rinoldi et al. (2019)
		Hyaluronate/PLGA/fibrin 3D scaffold loaded with human BMSCs was linked to cyclic strain bioreactor	Mechanical input stimulated expressions of tenogenic gene markers and pro-repair cytokines
			Ciardulli et al. (2020)
		Covalent crosslink-coordinated, physically reversible multicyclic hydrogel network from 25°C to 37 °C	The dynamically tunable mechanics of hydrogels induced cell spreading and differentiation of human MSCs
			Zhang et al. (2019)
		PEG-based hydrogels loaded with MSCs were stimulated under cyclic tensile strain	Upregulation of tendon-related genes, such as *Col III* and *TNC*
			Yang et al. (2012)
Biochemical	Hydrogel-based delivery	Heparin/fibrin-based hydrogels loaded with growth factor PDGF-BB and ADSCs	Sustained release of growth factors and cells to promote flexor tendon healing
			Manning et al. (2013)
		3D hydrogel scaffold carrying PLGA-NCs loaded with growth factor hGDF-5	Enhanced expression of tenogenic markers and collagen deposition of hWJ-MSCs
			Ciardulli et al. (2021)
		SSAD-derived hydrogel containing growth factors, IGF-1 and SDF-1	Increase in collagen fiber deposition and biomechanical properties of regenerated tendons. Suppression of peritendinous adhesion
			Dang et al. (2022)
		Tendon-specific ECM hydrogels loaded with ADSCs and growth factors IGF-1, PDGF-BB, and bFGF	The combination of growth factors could stimulate cellular proliferation within the gel
			Farnebo et al. (2017)
		Composite hydrogels loaded with Mg^2+^ and curcumin	The sustained release of Mg^2+^ and curcumin simultaneously was beneficial for rotator cuff healing by promoting regeneration of fibrocartilage tissues and organized collagen fibers
			Chen et al. (2021a)
		Composite hydrogel incorporating synthetic fibers and microgel-based PDGF-BB delivery	Recruitment of endogenous TPCs and induction of tenogenic differentiation in mouse Achilles tendon explants
			Kent et al. (2022)
		Thermosensitive hydrogel loaded with composites of KGN and MBGs	Promotion of fibrocartilage and bone regeneration through the bi-lineage induction of TDSCs. Enhanced mechanical property of the supraspinatus and humerus complex
			Huang et al. (2022)
		Fibrin hydrogels embedded with BMSCs-exos	Enhanced proliferation, migration and tenogenic differentiation of local TDSCs. The healing tendon exhibited improved collagen deposition and better mechanical properties
			Yu et al. (2020)
		Chitosan/beta-glycerophosphate/collagen hydrogel loaded with BMSCs-exos	Enhanced tendon-to-bone healing by promoting M2 macrophage polarization. Promotion of fibrocartilage regeneration at the tendon-bone interface and biomechanical properties
			Shi et al. (2020)
		ADSCs-exos-hydrogel complex	Higher mRNA expressions of tenogenic genes, more regular alignment of collagen fiber and muscle bundles, and improved biomechanical healing of rotator cuff tear
			Fu et al. (2021)
	Surface chemistry	Polyacrylamide gels functionalized with whole length fibronectin or collagen	BMSCs differentiation towards the tenogenic lineage was only observed on collagen substrates
			Sharma and Snedeker (2010)
		Synthetic thermosensitive hydrogels functionalized with collagen I	The survival, proliferation, and metabolic activity of TDSCs were improved by the incorporation of collagen I
			Yin et al. (2018)
		PEG-based hydrogels modified with bioactive peptides	Increasing concentration of integrin-binding peptides promoted the tenogenic gene expression of human MSCs
			Rehmann et al. (2016)
		Alginate hydrogels modified with RGD peptides	TPCs exhibited spread and fibroblastic-like morphology on RGD modified hydrogels, while cells were rounded on control hydrogels
			Marturano et al. (2016)
		Alginate hydrogel microspheres modified with RGD peptides	Dental derived MSCs encapsulated in RGD-coupled alginate microspheres exhibited higher expression levels of *Scx*, *Tnmd* and *Dcn*, and generated tendon-like organization
			Moshaverinia et al. (2014)
		Co-electrospun SF and GelMA sheet seeded with MSCs	Promotion of cell attachment and tenogenic differentiation of MSCs, and regeneration of damaged tendons
			Xue et al. (2022)

### 4.1 Biophysical factors of hydrogels for stem cells

**FIGURE 4:**
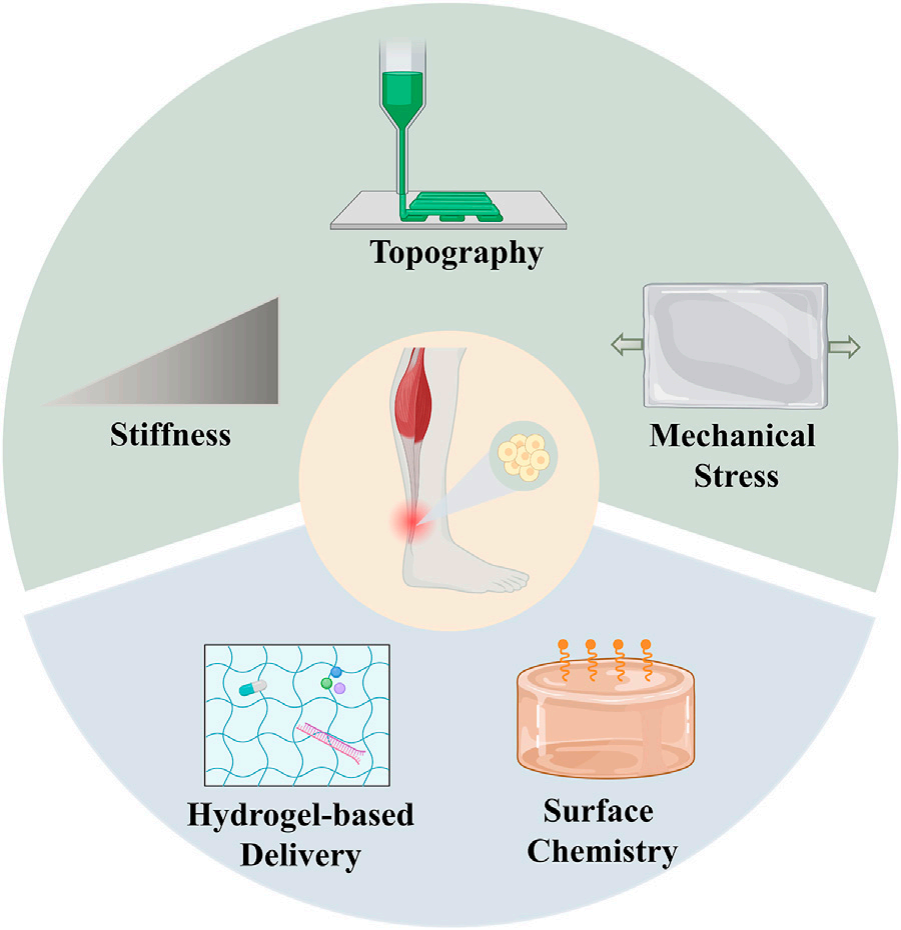
Strategies of hydrogels with tunable biophysical and biochemical factors for stem cell therapies in tendon repair. The upper part represents the biophysical factors of hydrogels, which include stiffness, topography and mechanical stress. The lower part represents the biochemical factors of hydrogels, which include hydrogel-based delivery and surface chemistry.

The direct interaction between stem cells and biomaterials has been considered to have a profound impact on the cell behaviors. The stem cells could directly sense the physical factors of biomaterials, such as the mechanical properties and surface topography (Martino et al., 2012; Lin et al., 2018; Donderwinkel et al., 2022). External mechanical loading has also been proven to motivate the tenogenic differentiation of stem cells. In this regard, there’s an emerging need for the design of hydrogels with tunable biophysical factors to regulate the fate of stem cells in tendon tissue engineering (Figure 4).

#### 4.1.1 Effect of material properties on stem cells

Substrate stiffness is one of the most common factors that stem cells sense in the ECM microenvironment. Previous researches suggested that substrate stiffness plays a pivotal role in the proliferation, migration and differentiation of stem cells (Engler et al., 2006; Konar et al., 2022). In terms of tendon tissues, substrate stiffness has been suggested to affect the tenogenic differentiation of stem cells (Islam et al., 2017). For instance, Sharma et al. evaluated the effects of substrates with a gradient of stiffness, ranging from 20 kPa to 80 kPa, on the differentiation of BMSCs. The BMSCs differentiated towards the tenogenic lineage on the collagen substrate. In addition, the expression of tendon-related genes peaked at the stiffness of 40 kPa (Sharma and Snedeker, 2010). Similarly, Rehmann et al. observed elevated expression levels of the tendon/ligament-associated genes, with the modulus of PEG-based hydrogel increasing from 10 to 50 kPa (Rehmann et al., 2016). However, in another study, the researchers cultured the TDSCs on the gelatin hydrogels with different matrix stiffness by varying the cross-linking level of gelatin. The results exhibited that increased matrix stiffness could promote the proliferation of TDSCs and the formation of F-actin stress fibers, while the tenogenic differentiation of TDSCs was inhibited (Liu et al., 2018). The above studies suggest that stem cells are sensitive to the changes in substrate stiffness, as only a narrow range of substrate stiffness would lead to tenogenic differentiation.

Tendon tissues are featured by their oriented anisotropic structure, which are composed of highly aligned collagen fibers. The collagen fibers further assemble into fascicles and the tendon unit, showing a hierarchical fibrillar arrangement. The anisotropic structures with hierarchical properties contribute to the high tensile strength of tendons. Therefore, researchers have been dedicated to designing novel biomaterials to mimic the topography of tendon tissues to guide cell contact, and regulate the alignment and differentiation of cells. An anisotropic nanocomposite hydrogel was fabricated through the mechanical strain generated by aligned iron oxide nanoparticles under magnetic field. The anisotropic hydrogel was demonstrated to guide the linear alignment of human TDSCs and enhance tenogenic differentiation in the absence of bioactive factors (Xu et al., 2021). Similar strategy employing external magnetic fields to manipulate the orientation of nanoparticle was reported by Echave et al. The anisotropic structure of gelatin hydrogel was fabricated by incorporation of cellulose nanocrystals and exposure to magnetic field. Human ADSCs were encapsulated in the anisotropic hydrogel, which exhibited a spindle-shape morphology and higher expression level of TNC (Echave et al., 2019). In order to mimic the three-dimensional (3D) architecture of tendon tissue, Yang et al. developed a multilayered composite scaffold composed of PCL fibers and mGLT hydrogel network. The ADSCs cultured on the multilayered scaffold were able to impregnate, elongate and align in the direction of fibers with enhancement in metabolic activity. PCR analysis showed upregulation of tendon markers when treated with tenogenic factor TGF-β3, indicating the loaded cells were able to respond to the external signals (Yang et al., 2016). An asymmetric hydrogel scaffold for tendon tissue engineering was developed from the natural polymer chitosan in another research (Chen et al., 2018). The asymmetric chitosan scaffold was composed of two different layers, a dense membrane layer and a loose spongy layer, in order to mimic the outer fibrotic layer and inner synovial layer of native tendon tissues. TDSCs were incorporated within the spongy layer with high porosity, which was beneficial for the cell proliferation and nutrition exchange. Additionally, the outer layer served as membrane for preventing tissue adhesion and providing mechanical support. TDSCs cultured within the chitosan scaffold exhibited significantly increased expression of tenogenic-related genes and proteins. *In vivo* studies conducted on rat tendon defect models demonstrated that the TDSC-seeded scaffold promoted tendon maturation and the regenerated tendons showed elevated post-operative tensile modulus and fewer adhesions.

Recent progresses in designing hydrogels with the technique of 3D printing allow the researchers to fabricate more precise and tunable structures, better mimicking the mechanical microenvironment of stem cells *in vivo* (O'Brien et al., 2014; Do et al., 2015). Ma et al. reported a composite hydrogel scaffold with multiscale structure for tendon tissue engineering. Calcium silicate (CS) nanowires were aligned in the direction of 3D printed direction in the 3D printed alginate composite hydrogels. The CS nanowires/alginate composite hydrogels were subsequently stretched to form multiscale fibers in the composite hydrogels. The composite hydrogels were capable of inducing the orderly arrangement of TDSCs and motivating their tenogenic differentiation to achieve functional tendon regeneration. *In vivo* study demonstrated that the composite hydrogel contributed to more aligned collagen matrix and better mechanical properties of healed tendons (Ma et al., 2022). Some researchers have progressed the application of 3D printing into 3D bioprinting by which cellular-based bioinks are applied to generate controlled constructs in a layer-by-layer manner. The emergence of 3D bioprinting technique offers an efficient strategy to attain homogeneous distribution of cells in the scaffolds (Murphy and Atala, 2014; Pati et al., 2016; Potyondy et al., 2021; Jiang et al., 2022). Jiang et al. combined the 3D bioprinting and melt electrospinning technologies to regenerate the tendon-to-bone interface. The 3D bioprinted hydrogels were encapsulated with three different cell lineages (tenogenic, chondrogenic, and osteogenic), which were differentiated from the autologous adipose-derived mesenchymal stem cells, to mimic the gradient structure and cell distribution of tendon-to-bone interface. The cell-laden 3D hydrogels were then stacked with 3D-printed PCL/poly lactic-co-glycolic acid (PLGA) scaffolds to guarantee the mechanical strength of the scaffolds. *In vivo* implantation of multilayered scaffolds demonstrated superior histological outcomes and collagen organization (Jiang et al., 2022). The combination of 3D bioprinted cell-laden hydrogels with certain synthetic materials provides a solution to the challenge of low mechanical strength in 3D bioprinting.

#### 4.1.2 Effect of mechanical stimulation on stem cells

The stem cells could also dynamically sense the mechanical stimulation *via* superficial mechanosensors, which further activate the intracellular signaling pathways to guide stem cell fate (Morris et al., 2016; Sun et al., 2022). It is generally considered that proper mechanical loading contributes to the tenogenic differentiation of stem cells, while aberrant mechanical loading would lead to non-tenocyte lineage differentiation. For instance, dynamic mechanical forces have been demonstrated to regulate tenogenic differentiation (Schiele et al., 2013; Lin et al., 2018; Wang et al., 2020a). Thus, modulating the mechanotransduction between stem cells and hydrogels is of great importance.

Park et al. used the gel aspiration-ejection method to fabricate aligned dense collagen (ADC) hydrogel scaffolds and human BMSCs were further seeded along the scaffolds. The ADCs showed anisotropic structures that mimic the structure and strength of tendon. The MSCs-seeded ADC hydrogels were mechanically stimulated uniaxially for 48 h under static strain or cyclic rest. Compared to the controls, which were free-floating, the static strain and cyclic rest ADC hydrogels exhibited elevated expressions of tenogenic marker, *Scx*, and lower expressions of osteogenic and chondrogenic markers, *RUNX2* and *aggrecan* (Park et al., 2022a). This MSCs-seeded ADC hydrogel was expected to be implanted to form tendon-like tissues after short-term mechanical stimulation. In another study, Rinoldi et al. employed the wet-spinning technique to fabricate highly aligned GelMA-alginate yarns which were loaded with human BMSCs. After static mechanical stretching was subjected to the cell-loaded hydrogel yarns, the cells were observed to be aligned along the direction of stretching, with higher expression levels of collagen I and III (Rinoldi et al., 2019). The combination of mechanical stretching with the highly aligned internal structure of the hydrogel yarns provided mechanical microenvironment similar to tendon tissue.

Previous efforts in regulating the biophysical factors for tenogenic differentiation of stem cells have been focusing on using static biomaterials, while applying external mechanical stimulation. However, the *in vivo* mechanical microenvironment around the stem cells appears to be highly dynamic. The native ECM, surrounding cells and other bioactive factors persistently undergo changes and the stem cells could sense and respond to the dynamic changes correspondingly (Morris et al., 2016; Ma et al., 2018; Zhang et al., 2019; Park et al., 2022b). Therefore, the scope of interest in designing biomaterials has shifted from simple and bioinert materials to biomimetic materials with dynamic behaviors. The research conducted by Ciardulli et al. compared the influence of static conditions and cyclic strain conditions on the tenogenic differentiation of BMSCs (Ciardulli et al., 2020). The BMSCs were distributed within the hyaluronate/PLGA/fibrin 3D scaffold. The hyaluronate braided band was linked to cyclic strain bioreactor to generate a shear stress value estimated at 9 × 10^−2^ Pa within the scaffold. RT-PCR data exhibited significantly elevated expressions of tenogenic gene markers and pro-repair cytokines under dynamic culture. In a recent study, the researchers encapsulated the human MSCs spheroids in thermo-responsive hydrogels, which were constructed by interpenetrating poly (N-isopropylacrylamide-co-2-hydroxyethyl methacrylate) nanogels to GelMA network. By altering the temperature from 25 °C to 37 °C, the thermo-responsive hydrogels could transform temperature into changes in stiffness. The dynamically tunable mechanics of hydrogels offered a platform to guide the differentiation of human MSCs (Zhang et al., 2019). In another study, MMP-sensitive PEG-based hydrogels loaded with MSCs were into tensile culture bioreactor. The metalloproteinases produced by cells induced degradation of the hydrogel matrix, which allowed the spreading of MSCs. Meanwhile, the cyclic tensile strain was demonstrated to induce upregulation of tendon fibroblast-related genes, including *Col-III* and *TNC* (Yang et al., 2012). The advances in material science have made it possible for researchers to mimic the dynamic interactions between stem cells and surrounding microenvironment, offering a platform to regulate the fate of stem cells in a higher dimension.

### 4.2 Biochemical factors of hydrogels for stem cells

Compared to the effect of biophysical factors, biochemical factors have been widely studied and are considered to play a pivotal role in regulating the fate of stem cells. After the delivery of stem cells to the site of tendon injury, the ratio of cells surviving is relatively low and the fate of the stem cells could hardly be monitored, leading to massive cell death or non-tenogenic differentiation (Lui, 2015). In this regard, it was purposed that simply delivering stem cells might not be sufficient to achieve ideal tendon repair, while the addition of proper biochemical factors offer solutions to enhance the expansion of stem cells and stimulate tendon regeneration (Gulotta et al., 2009; Chen et al., 2022) (Figure 4).

#### 4.2.1 Hydrogels loaded with biochemical factors

Biochemical factors, such as growth factors, small bioactive molecules and genetic regulators, have been applied to modulate the proliferation and differentiation of stem cells through different signaling pathways (Docheva et al., 2015; Li et al., 2021b). As previously indicated, the applications of these biochemical factors are hampered by issues including burst release and fast clearance at the delivery site. To this end, researchers have shifted their focus to the combination of biochemical factors and hydrogels with loading capacity.

Several growth factors, which include IGF-1, TGFβ, platelet-derived growth factor (PDGF), and bFGF, have been found to be essential in the process of both tendon development and tendon repair (Molloy et al., 2003; Farnebo et al., 2017). The growth factors could be integrated into the hydrogels to extend their duration of action to recapitulate a favorable microenvironment for the stem cells. Manning et al. reported a novel scaffold for tendon repair, which was capable of delivering growth factors and stem cells in a controllable manner (Manning et al., 2013). The heparin/fibrin-based delivery system (HBDS) was loaded with growth factor PDGF-BB and ADSCs. The hydrogel was then hybridized with the electrospun PLGA nanofiber mats to obtain mechanical strength, together forming a stacking structure on top of each other. Compared to the fibrin scaffold alone, the HBDS demonstrated more sustained delivery of PDGF-BB. Furthermore, *in vivo* studies showed that the delivered cells could survive for at least 9 days after the operation. In another research, Ciardulli et al. designed a functionalized 3D biomimetic scaffold for promoting tenogenic differentiation of human mesenchymal stem cells collected from Wharton’s Jelly (hWJ-MSCs) (Ciardulli et al., 2021). The scaffold was composed of a braided hyaluronate elastic band and a fibrin hydrogel, which was loaded with poly-lactic-co-glycolic acid nano-carriers (PLGA-NCs) carrying human Growth Differentiation factor 5 (hGDF-5). The braided hyaluronate elastic band was connected to the cyclic strain bioreactor to impose a cyclic deformation to the scaffold, while the PLGA-NCs provided controlled delivery of growth factor hGDF-5 within the scaffold. The dynamic culture environment of the scaffold was considered to be essential for the controlled release of growth factors by PLGA-NCs. *In vitro* experiments demonstrated that, compared to hGDF-5 supplemented culture medium, the hWJ-MSCs distributed within the 3D scaffold exhibited enhanced expression of tenogenic markers and collagen deposition. Instead of the combination of hydrogel with exogenous growth factors, Dang et al. reported hydrogel derived from the skin secretion of amphibian, which contained abundant natural growth factors, such as IGF-1 and stromal cell-derived factor 1 (SDF-1). The released growth factors could bring benefits to tendon healing by promoting the migration and proliferation of TDSCs. *In vivo* experiments indicated that the hydrogel gave rise to functional tendon repair by triggering collagen deposition, cell proliferation and inhibiting peritendinous adhesion (Dang et al., 2022). Compared to a single growth factor, it has been purposed that the combination of multiple growth factors would be superior in imitating the complicated *in vivo* environment (Molloy et al., 2003; Indrawattana et al., 2004; Gaspar et al., 2015). For instance, Farnebo et al. found that the addition of growth factors, including FGF, IGF, and PDGF, were able to improve the survival of ADSCs seeded to the tendon-specific ECM hydrogel. The incorporation of the combined growth factors with ADSCs exhibited a synergistic effect on the cell recruitment and repopulation of the gel (Farnebo et al., 2017). A composite hydrogel system designed by Kent et al. combined the biochemical factors with physical factors so as to promote the recruitment of tendon progenitor cells (TPCs) for tendon repair. The researchers first identified a type of chemokine, PDGF-BB, which induced TPC migration into the composite hydrogel. In order to achieve the gradual release of PDGF-BB from the hydrogel, methacrylated heparin (HepMA) was incorporated into the vinyl sulfonated dextran hydrogel. HepMA governed the release rate of the PDGF-BB through reversible affinity interactions with the soluble factors. Synthetic, cell adhesive synthetic fibers were further incorporated with the hydrogel to provide anisotropic structures, which were suitable for promoting cell migration into the constructs. Mouse Achilles tendon explants were encapsulated in the hydrogel system to testify the effect of TPC recruitment. The hydrogel system significantly enhanced TPCs recruitment and induced tenogenic differentiation of TPCs (Kent et al., 2022).

Other efforts have combined hydrogels with metallic ions and bioactive molecules. In a recent study, Huang et al. developed injectable bioactive thermosensitive hydrogel loaded with composites of kartogenin (KGN) and mesoporous bioactive glass nanoparticles (MBGs) (KGN@MBGs) for chronic rotator cuff repair. After injection into the injury sites, the hydrogel had the ability to self-heal and became solid in response to temperature changes, which developed a suitable environment for the sustained release of KGN@MBGs *in situ*. The released KGN@MBGs were demonstrated to enhance the *in vitro* osteogenesis and chondrogenesis process of TDSCs. This bioactive thermosensitive hydrogel was also tested in rabbit chronic rotator cuff tears model and exhibited significant promotion in bone layer and fibrocartilage regeneration. Furthermore, the composite hydrogel also brought benefits to the mechanical properties of humerus-tendon complex (Huang et al., 2022). Chen et al. developed a composite hydrogel with the capacity for sustained release of Mg^2+^ and curcumin for rotator cuff healing. The released curcumin exhibited anti-inflammatory effects by protecting BMSCs from oxidative stress. At the same time, the loaded Mg^2+^ promoted the recruitment of MSCs. The composite hydrogel implanted in model rats improved tendon collagen fiber organization and biomechanical performance of the regenerated rotator cuff, therefore showing great potential in tendon tissue engineering (Chen et al., 2021a).

The paracrine effects of stem cells in tissue regeneration have been the subject of extensive researches in recent years (Baraniak and McDevitt, 2010; Ratajczak et al., 2012). Among all the secretome of stem cell origin, exosomes secreted from stem cells have attracted research interests. Exosomes facilitate intercellular communication *via* a paracrine pathway and the sustained release of exosomes from hydrogel-based materials may offer a promising strategy for tendon repair (Villarroya-Beltri et al., 2014; Vizoso et al., 2017; Tang et al., 2021). Yu et al. explored the promoting effects of BMSCs-derived exosomes (BMSC-exos) on TDSCs and embedded them into fibrin gels. The release dynamics of BMSC-exos from the gels showed that the BMSC-exos could be preserved at the injury site for at least 2 weeks. *In vivo* study in a murine patellar tendon injury model exhibited that the BMSC-exos containing fibrin gels could enhance tendon regeneration by promoting the proliferation of TDSCs in the early phase of tendon repair (Yu et al., 2020). In another study embedding the BMSCs in chitosan/b-glycerophosphate/collagen hydrogels, the researchers found that the release of BMSC-exos could also enhancing the tendon-bone healing by regulating the inflammatory microenvironment (Shi et al., 2020). Apart from the exosomes obtained from BMSCs, ADSCs-derived exosomes (ADSCs-exos) have attracted extensive interest, as ADSCs are easy to obtain from the adipose tissues (Chen et al., 2021b; Lyu et al., 2022). Previous studies have revealed that ADSC-exos could improve tendon healing in multiple ways, including promoting angiogenesis, stimulating the activity of tenocytes and decreasing inflammatory responses (Chen et al., 2021b; Fu et al., 2021; Mazini et al., 2021; Lyu et al., 2022). Fu et al. developed an adipose-derived stem cell exosome-hydrogel complex (EHC) for rotator cuff repair. *In vitro* experiments demonstrated that exosomes derived from ADSCs could promote the proliferation and differentiation of TDSCs. To examine the effect of EHC on tendon repair, the researchers implanted the EHC into injured rotator cuff muscles of rats. Histological analysis exhibited improved regeneration of collagen fibers and muscle bundles over standard controls. Their data revealed that ADSCs-exos could upregulate the mRNA expression level of tenogenic genes (*TNC*, *TNMD* and *Scx*) (Fu et al., 2021).

The applications of biochemical factors in tendon repair are limited by difficulty in retaining at the injury sites and the hypocellularity of tendon tissues, whereas the applications of stem cell-based therapies are constrained by the absence of sufficient signals and microenvironments for the proper proliferation and differentiation of the implanted cells. Loading the biochemical factors and stem cells together in the functionally-adapted hydrogels could complement each other’s advantages, representing a promising approach to encourage tendon regeneration.

#### 4.2.2 Surface chemistry modification of hydrogel

It has been postulated that the survival, proliferation, and differentiation of stem cells depend on their interactions with tendon ECM through cell adhesion ligands (Linsley et al., 2013; Clements et al., 2016; Li et al., 2018b; Ryan and Zeugolis, 2020). The tendon microenvironment could be mimicked by employing hydrogels fabricated from naturally derived proteins, which consist of sufficient cell adhesion ligands. However, in most synthetic hydrogels, a lack of adhesive molecules for encapsulated cells would lead to undesired cell death (Tan et al., 2005; Li et al., 2018b; Cao et al., 2021). Therefore, hydrogel-based therapies for tendon regeneration may benefit greatly from the incorporation of native ECM molecules or adhesion peptides in hydrogel fabrication to enhance cell adhesion.

Collagen and fibronectin have been the most common proteins in tendon tissues to regulate cell adhesion (Miyamoto et al., 1998; Giancotti and Ruoslahti, 1999). Sharma et al. functionalized the hydrogel substrates with whole length fibronectin or collagen type I of different densities. Analysis of cell attachment showed that BMSC cell attachment increased in proportion to the increase in bulk ligand density. Surprisingly, the researchers found that BMSCs differentiated towards different lineages depending on the type of the functionalized ligands. Tenogenic differentiation of BMSCs was only observed on collagen substrates in a relatively small range of substrate compliance, with the upregulation of *Scx*, *Tnc*, *Tnmd* and *Col III* expressions (Sharma and Snedeker, 2010). In a similar study, the researchers functionalized the synthetic thermosensitive hydrogels with the addition of collagen I. Compared to the non-functionalized hydrogels, the incorporation of collagen I significantly improved the survival, proliferation, and metabolic activity of TDSCs, thus circumventing the poor biocompatibility of synthetic hydrogels (Yin et al., 2018).

Another commonly utilized approach to modify the surface chemistry of biomaterials is to combine peptide sequences which are integrin ligands (Stowers, 2022). Hydrogels functionalized with cell adhesion peptides have been extensively applied in the differentiation of stem cells toward tenogenic, osteogenic, and adipogenic lineages (Wang et al., 2013; Moshaverinia et al., 2014; Zhang et al., 2021b). Moshaverinia et al. designed a promising hydrogel-based delivery system for tendon regeneration, in which dental derived MSCs were encapsulated in alginate hydrogels coupled with RGD peptide. Compared to the non-RGD coupled hydrogels, the presence of RGD peptide significantly improved the viability of the encapsulated MSCs. In addition, the MSCs encapsulated in the RGD-containing alginate hydrogels exhibited higher levels of tendon-related genes, such as *Scx*, *Tnmd* and *Dcn*. The researchers further loaded the RGD-coupled alginate hydrogels with growth factor TGF-β3. The synergistic effect of cell adhesion peptide and growth factor significantly facilitated the tenogenic differentiation of MSCs *in vitro* and tendon tissue regeneration *in vivo* (Moshaverinia et al., 2014). However, another study functionalizing the RADA hydrogel with RGD peptide showed opposite results. The RGD-containing hydrogel did not exhibit significant beneficial effect on TDSCs over the non-RGD coupled one (Yin et al., 2020). Therefore, further investigations are required to determine the type, density and spatial location of cell adhesion peptides suitable for tendon tissue engineering. GelMA, an extensively investigated synthetic hydrogel, has been suggested to promote cell attachment and proliferation owing to its intrinsic RGD motifs (Yue et al., 2015). On this basis, Xue et al. combined the GelMA with silk fibroin (SF) nanofibers to fabricate a scaffold seeded with MSCs. The SF nanofibers introduced better mechanical properties into the scaffold. The composite scaffold provided a desirable microenvironment for the adhesion and migration of MSCs, and induced their tenogenic differentiation at the same time. *In vivo* studies conducted in the rat Achilles tendon injury model showed wel l-aligned and densely packed regenerated tendons in the group treated with MSC-seeded scaffold (Xue et al., 2022). In a word, the addition of cell adhesion peptides into the composite hydrogel, such as RGD, could optimize the biocompatibility of the biomaterials and provide the ideal milieu for stem cell viability and differentiation.

## 5 Hydrogel and macrophage

Following tendon injury, macrophages infiltrate into the site of injured tendon and modulate local inflammatory and healing process, playing an important role in the process of tendon repair (Chisari et al., 2020; Russo et al., 2022a). Macrophages can be differentiated into M1 and M2 phenotypes, which present different biological functions. M1 macrophages are mainly responsible for secreting multiple inflammatory factors and stimulating inflammatory responses, while M2 macrophages are in charge of reducing inflammation and promoting tissue repair (Murray Peter et al., 2014; Sunwoo et al., 2020). As previously mentioned, M1 macrophages are the primary drivers of the initial inflammatory phase after tendon injury, which participate in the release of proinflammatory cytokines and phagocytosis of tissue debris and apoptotic cells (Sunwoo et al., 2020). A previous study revealed that M1 macrophages rapidly accumulated in the newly formed tendon tissues in 3 days after surgery tear and repair in the murine Achilles tendon, while the concentration of M2 macrophages significantly increased by 28 days and became the predominant macrophage phenotype (Sugg et al., 2014). M2 macrophages are considered to be involved in promotion of fibroblast proliferation and new tissue deposition, which are pivotal for the regeneration and remodeling of tendon tissues (de la Durantaye et al., 2014; Sunwoo et al., 2020).

**FIGURE 5:**
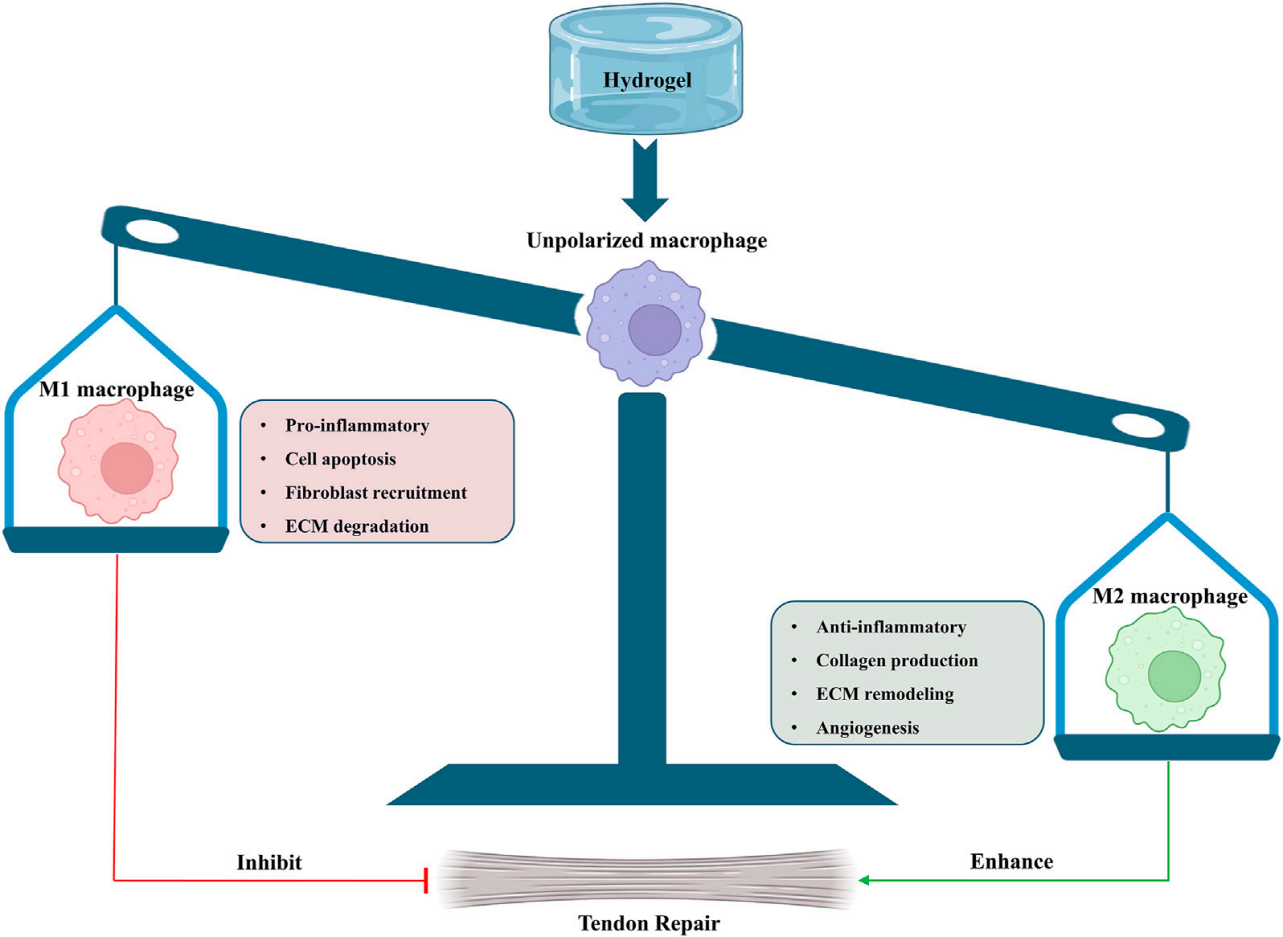
Effects of functionally-adapted hydrogels on macrophage polarization in tendon repair.

Recent researches have been focusing on the switch of M1 to M2 macrophage polarization to promote functional tendon repair. The M2 macrophages are expected to support tendon repair by mitigating inflammatory response and producing extracellular matrix components (Rőszer, 2015; Sunwoo et al., 2020). Persistent activation of M1 macrophages is considered to contribute to excessive inflammatory response and recruitment of fibroblasts, which result in formation of scar tissue and tendon adhesion (Shapouri-Moghaddam et al., 2018; Cui et al., 2019; Yang et al., 2021). The transition of macrophages from M1 to M2 would reduce the local inflammatory response by releasing several anti-inflammatory cytokines, such as IL-10 and TGF-β (Murray et al., 2014; Shapouri-Moghaddam et al., 2018; Shi et al., 2020). However, anti-inflammatory drug administrations for the first 5 days in tendon transection model have been proven to have a detrimental effect on tendon repair, suggesting that inflammation during the initial phases of tendon repair could be beneficial (Virchenko et al., 2004). Additionally, it has been proposed that the overactivation of M2 macrophages could also lead to pathological fibrosis (Ackerman et al., 2017). Instead of promoting the polarization of macrophages into certain phenotype, it is more important to identify the optimal balance between inflammation and regeneration in tendon repair and activate the macrophage polarization at proper stage (Figure 5).

Due to their exceptional biocompatibility, tuneability and drug loading capacity, the hydrogels are attractive immunomodulatory biomaterials in tendon repair. Most researches have utilized the hydrogels as carriers to deliver biologics and drugs to modulate the behavior of the macrophages and the conversion between M1 and M2 phenotypes (Table 3). Yang et al. applied hydrogels loaded with cyclooxygenases (COX) siRNA/nanoparticles with the capacity to modulate macrophage polarization in tendon repair. *In vivo* study in the injured murine Achilles tendon demonstrated that the siRNA/nanoparticle hydrogel complexes could improve the healing strength of injured tendons and decrease inflammatory reactions, especially in the early stage of tendon healing. The therapeutic potential of COX siRNA/nanoparticles loaded hydrogels was attributed to the M1 to M2 conversion after inhibition of COX. The gradually released COX siRNAs could increase the proportion of M2 macrophages *in vitro*, which were assumed to inhibit inflammation and enhance tissue repair. The hydrogel loaded with COX siRNA/nanoparticles was suggested to increase the ultimate strength of the tendons in the rat Achilles tendon injury model (Yang et al., 2022). In another study, Xu et al. designed an injectable hydrogel composed of bioactive glass (BG) and sodium alginate (SA) to enhance tendon healing. *In vivo* study exhibited that the addition of BG not only alleviated the accumulation of M1 macrophages induced by SA hydrogel, but also increased the polarization of macrophages toward M2 phenotype in the early stage of tendon healing. The increased amount of M2 macrophages further promoted the angiogenesis in the granulation tissue during tendon healing, which was attributed to the pro-angiogenic factors secreted by M2 macrophages. *In vivo* studies demonstrated that BG/SA hydrogel reversed the pathological morphological changes of Achilles tendon, and enhanced the biomechanical properties of reconstructed tendons, such as ultimate load, failure stress and tensile modulus. Therefore, the BG/SA hydrogel represented a promising biomaterial for tendon repair *via* modulating inflammatory reactions and addressing the hypovascularity state of tendon tissues (Xu et al., 2022b). Freedman et al. reported a tough adhesive hydrogel with high drug-loading ability for tendon tissue engineering. The hydrogel was capable of achieving the sustained and local release of anti-inflammatory drug, triamcinolone acetonide (CORT). The CORT loaded hydrogel regulated the immune reactions in tendon repair, which was demonstrated by an increase in M2 macrophage phenotype (Freedman et al., 2022b). Wang et al. reported a gelatin hydrogel dressing, which contained anti-inflammatory drugs celecoxib and Fe_3_O_4_ nanoparticles for the treatment of tendon injuries. Under the stimulation of pulsed electromagnetic field, the combined effect of Fe_3_O_4_ nanoparticles shaking and generated heat could loosen the hydrogel network and accelerate the release of celecoxib. *In vivo* studies of rat Achilles tendon rupture models illustrated that the hydrogel dressing contribute to increase in M2 macrophages and reduction in inflammatory response during tendon repair. Groups treated with the hydrogel dressing showed better outcomes CatWalk gait analysis system (Wang et al., 2020b). Hydrogel-based delivery strategies allow for the biologics and drugs to distribute evenly around the injured tendon and provide an approach to precisely control the degree and timing of macrophage polarization.

**TABLE 3:** The effect of immunomodulatory hydrogels on macrophage regulation in tendon repair.

Approach	Outcome
COXs siRNA/nanoparticle loaded hydrogel	Increase in M1 to M2 switch of macrophages and enhanced mechanical strength of repaired tendons
	Yang et al. (2022)
Injectable hydrogel composed of BG and SA	Increase in angiogenesis and M1 to M2 switch of macrophages. The injectable hydrogel reversed the pathological changes of injured tendons and enhanced the mechanical properties of repaired tendons
	Xu et al. (2022b)
Tough hydrogels loaded with CORT	Increase in cellularity and M2 macrophage polarization
	Freedman et al. (2022b)
Hydrogel combined with celecoxib and Fe_3_O_4_ nanoparticles	Coordinated drug release of celecoxib under the stimulation of magnetic field. Increase in M2 macrophages and reduction in inflammatory response in tendon injury sites. Better outcomes of CatWalk gait analysis
	Wang et al. (2020b)
Self-healing HA hydrogel	Suppression of macrophage recruitment and M2 macrophage polarization to inhibit tendon adhesion
	Cai et al. (2022)

The biocompatibility of hydrogels also plays an essential role in modulating the behavior of macrophages. Despite their excellent biocompatibility, innate and adaptive immune response would be triggered after hydrogel introduction into the injury site. The consequent acute or chronic inflammatory response could further lead to a cascade of events called foreign body reaction (FBR) and development of tissue adhesion (Anderson, 2001; Russo et al., 2022c; Wang et al., 2022). After the biomaterial implantation, the inflammatory-related proteins, mainly blood and plasma proteins, adhere to the surface of the implanted hydrogels. The circulating leukocytes, monocytes and macrophages are attracted around the implanted sites and surrounding tissues, which subsequently start to secrete pro-inflammatory and angiogenic cytokines. The secreted cytokines are able to recruit tissue repair cells and ultimately contribute to the formation of dense fibrotic capsules around the implanted biomaterials (Anderson et al., 2008; Yan et al., 2019; Gori et al., 2021; Russo et al., 2022c). Therefore, the ideal functionally-adapted hydrogels for tendon repair should be designed to promote tendon healing without inducing excessive pro-inflammatory responses. For instance, the fabrication of hydrogels with self-healing properties reduced the non-infectious inflammation induced by broken fragments generated by the rupture of hydrogels. Compared to non-self-healing hydrogel, the self-healing hydrogel recruited fewer macrophages to the injury site and decreased M2 macrophage polarization to inhibit tendon adhesion (Cai et al., 2022).

## 6 Conclusion and future directions

Although enormous researches have provided in-depth understanding on the structure and component of tendon tissues and changes initiated during the process of tendon repair, tendon injuries still pose a clinical challenge due to lack of adequate cellularity and vascularity for optimal tendon repair. Current therapeutic strategies for tendon injuries, which include anti-inflammatory drugs, injection of growth factors or surgeries, have difficulties in restoring the original structures and functions of tendons to pre-injury states. The rapid advances in tissue engineering have brought new possibilities in the treatment of tendon injuries. Various biomaterials have been developed and investigated as potential substitutes for enhancement of tendon repair. Thanks to their exceptional plasticity and biocompatibility, the application of hydrogels as scaffolds or carriers continue to attract extensive research interest.

Despite of wide application of hydrogels in biomaterials, the conventional hydrogels tend to possess low mechanical strength, which severely restricts their applications in load-bearing tissues, such as tendons or ligaments. To overcome this issue, different strategies have been proposed to gain hydrogels with suitable mechanical properties for tendon repair, including interpenetrating hydrogels, hybridization with other polymers or nanocomposites and self-healing hydrogels. Combining hydrogels with the fibers represents another promising approach, in which the reinforced fibers provide favorable mechanical strength while the hydrogels mimic the properties of native ECM. Acting as the carriers of bioactive molecules, drugs and cells, the degradation behavior of hydrogels should also be taken into consideration. Apart from previous methods to control the degradation of hydrogels mainly through modifying the structures and components of hydrogels, stimuli-responsive hydrogels represent another promising field of research. However, it is still a challenge to determine the mechanical strength of hydrogels suitable for tendon repair and how to integrate the mechanical properties with other material properties. Current studies generally concentrate on modifying the degradation rate to achieve controllable delivery of drugs or biologics, while the decrease of mechanical strength is somehow ignored. Also, it is difficult to visualize the *in vivo* degradation of hydrogels and observe how hydrogels respond to the complicated microenvironment after tendon injury.

Regeneration of tendon tissues and formation of fibrosis or adhesion during tendon repair has been suggested to be manipulated by multiple cell populations, such as stem cells and macrophages. Due to insufficient knowledge on the roles that different cell types play in tendon repair, current treatments could hardly achieve desirable clinical outcomes. Despite various attempts in designing functionally-adapted hydrogels to regulate cell behaviors for tendon repair, the understanding of the process of tendon repair and the mechanism behind the interactions between cells and biomaterials remain elusive.

In recent decades, varieties of biomaterials have been designed with the aim of regaining full tendon functions. Even if hydrogels have demonstrated superior potentials, it would inevitably meet limitations in the complicated and prolonged process of tendon repair. Fabricating therapeutics strategies by combining hydrogels with diverse materials or technologies would be necessary to make the best use of the advantages, while overcoming the drawbacks of each alone. In addition, investigating the key cells and pathways involved in tendon repair, and linking them to the material properties of hydrogels represent another major topic in tendon tissue engineering. In spite of the great progress achieved in hydrogel research, the clinical translation of the related products has fallen short of expectations. The bulk of hydrogel-based strategies discussed in this review were only assessed *in vitro* or animal models, the post-implantation validity and safety of hydrogels, complexities in synthesis, and their interaction with surrounding tissues remain doubtful. With the expanding arsenal of materials, advances in understanding tendon repair mechanisms and cooperations between researchers from different fields, we believe that better biomaterials would be obtained to attain functional tendon regeneration and repair.

## Glossary

ECM: Extracellular matrix
TDSCs: Tendon-derived stem cells
bFGF: Basic fibroblast growth factor
TGF-β: Transforming growth factor beta
IGF-1: Insulin-like growth factor-1
PHEA-API: poly (2-hydroxyethyl aspartamide) modified with aminopropyl imidazole
PG: Polyglycerol-functionalized reduced graphene oxide
PMoS2: polyglycerol-functionalized molybdenum disulfide
GelMA: Gelatin methacryloyl
ADSCs: Adipose-derived stem cells
PCL: Poly-ε-caprolactone
mGLT: Methacrylated gelatin
UHMWPE: Ultrahigh molecular weight polyethylene
SSAD: *Andrias davidianus*
HA: Hyaluronic acid
PPZ: poly(organophosphazene)
PEG: Polyethylene glycol
OPF: Oligo(poly(ethylene glycol) fumarate)
DTT: Dithiothreitol
MSCs: Mesenchymal stem cells
MMP-2: Metalloproteinase-2
ROS: Reactive oxygen species
PTK: Poly(thioketal)
AECs: Amniotic epithelial cells
BMSCs: Bone marrow mesenchymal stem cells
3D: Three-dimensional
CS: Calcium silicate
PLGA: Poly lactic-co-glycolic acid
ADC: Aligned dense collagen
PDGF: Platelet-derived growth factor
HBDS: Heparin/fibrin-based delivery system
hWJ-MSCs: Human mesenchymal stem cells collected from Wharton’s Jelly
PLGA-NCs: Poly-lactic-co-glycolic acid nano-carriers
hGDF-5: Human Growth Differentiation factor 5
SDF-1: Stromal cell-derived factor 1
TPCs: Tendon progenitor cells
HepMA: Methacrylated heparin
KGN: Kartogenin
MBGs: Mesoporous bioactive glass nanoparticles
BMSC-exos: Bone marrow mesenchymal stem cells-derived exosomes
ADSC-exos: Adipose-derived stem cells-derived exosomes
EHC: Exosome-hydrogel complex
RGD: Arginine-glycine-aspartate
SF: Silk fibroin
COX: Cyclooxygenases
BG: Bioactive glass
SA: Sodium alginate
CORT: Triamcinolone acetonides
